# Processing poor quality ram semen by colloid centrifugation prior to freezing: a proof-of-concept study

**DOI:** 10.3389/fvets.2026.1946483

**Published:** 2026-10-09

**Authors:** Jane M. Morrell

**Affiliations:** 1 Clinical Sciences, Swedish University of Agricultural Sciences, Uppsala, Sweden; 2 CRESCO, Centre for Embryology and Healthy Development, Department of Biotechnology, University of Inland Norway, Hamar, Norway

**Keywords:** cryodamage, Ovicoll, Semen cryopreservation, sheep artificial insemination, Single Layer Centrifugation

## Abstract

**Introduction:**

The sheep breeding season is short in some breeds, restricting the availability of rams for semen collection as they are required for natural mating. Therefore, cryopreservation may need to be done before the start of the breeding season, when semen quality may be sub-optimal. Poor quality samples do not show good cryosurvival. Colloid centrifugation, particularly Single Layer Centrifugation (SLC), was shown previously to select the most robust spermatozoa from an ejaculate. The objective of the present study was to prepare sub-optimal ram ejaculates by scaled-up SLC to determine if sperm quality could be improved sufficiently for cryopreservation, in a proof-of-concept trial.

**Methods:**

Semen was collected from a ram during the month preceding the breeding season. The first ten ejaculates were discarded because of sub-optimal motility (< 70% motile in raw semen). Five further sub-optimal ejaculates were processed by SLC. The semen was extended in warm Andromed (30 °C) to 100 x 10^6^ spermatozoa/ml; 20 ml of extended semen were layered over 15 ml Ovicoll in a 50 ml tube and centrifuged for 20 min at 300 g. The sperm pellet was resuspended in Andromed (18 °C) at 500 x 10^6^ spermatozoa/ml before loading into plastic straws. The straws were cooled for 2 h at 6 °C and were then frozen in a programmable freezing machine. After storing for 24 h in liquid nitrogen, post-thaw motility was evaluated subjectively. Samples with post thaw motility ≥50% were used for artificial insemination.

**Results:**

Initial motility in the 5 ejaculates was 20-50%. After SLC, motility was 80%−90%, while post-thaw motility was 50% in all samples. Thawed semen was deposited vaginally in oestrous ewes (22), producing 46 lambs in total, of which 4 were still-born.

**Conclusion:**

Ram semen can be processed by SLC with Ovicoll to improve sperm cryosurvival.

## 1 Introduction

Sheep are short-day breeders with a defined breeding season, which can be short (a few months) in some breeds (1). Most sheep breeding globally is carried out by natural mating but for sheep owners wishing to improve the genetics of their flocks while maintaining biosecurity, artificial insemination (AI) is the reproductive biotechnology of choice (2). Results with fresh semen are generally good (>70% pregnancy rate), although frozen semen is less efficient, with a pregnancy rate of 30%−60% (3).

Cryopreserving ram semen presents challenges. The ejaculate typically consists of a small volume of semen with a high sperm concentration (4), making comparison of different cryomedia or cooling rates difficult. Ram spermatozoa exhibit susceptibility to cryo-induced damage (5), reducing their ability to reach the oocyte after artificial insemination. The ratio of cholesterol to phospholipid in the sperm plasma membrane is believed to be lower in sheep than other species (6), resulting in a greater cold -shock sensitivity. In addition, polyunsaturated and saturated fatty acids bound to membrane phospholipids contribute to this sensitivity by influencing membrane stability at low temperatures (7). Typically, only approximately 50% of spermatozoa survive the process of cryopreservation (8).

Various protocols exist for freezing ram semen but the pregnancy rate following vaginal deposition of thawed semen is low, as already mentioned (3). The cervix of the ewe is tightly folded, making passage of a catheter without causing damage extremely difficult (9). However, thawed spermatozoa are fertile if placed close to the oocytes, e.g., if introduced into the oviducts via laparoscopy (10), and are able to undergo the acrosome reaction and fertilize normally once in the vicinity of the oocytes. The ability to carry out these normal functions suggest that thawed spermatozoa have difficulty in passing through the cervix. Attempts to bypass the cervix in a non-traumatic manner have not been universally successful, although in some breeds it is possible to pass an insemination device a short way into the cervix (11).

Research on cryopreservation protocols has focused mainly on various supplements to the cryopreservation medium to mitigate cryodamage, e.g., antioxidants to preserve membrane integrity (12). However, although post-thaw motility may be increased by these additives, pregnancy rates show little or no improvements. Therefore, there is still much to be done before apparent success in the laboratory can be translated into success in the field.

A further problem with cryopreservation is that the ejaculate must be of suitable quality initially to deliver a usable post-thaw product. When semen is collected outside the normal breeding season, sperm quality may be poor, resulting in non-functional samples post-thaw. It takes approximately 6–8 weeks from the initiation of testicular activity in response to decreasing day length to achieve optimum sperm quality (13). In the present study, semen was collected from rams for freezing in the month prior to the presumed start of the breeding season because the rams were needed for natural mating in their home flocks once the season commenced. Although it was anticipated that sperm quality might be sub-optimal in the beginning of the semen collection period, it was thought that there would be an improvement as the collection period progressed that would enable semen to be frozen.

A method for improving sperm quality by selecting robust spermatozoa using colloid centrifugation has been used extensively for stallion semen (14). The method functions in the same manner as a density gradient but utilizes only one layer of colloid (Single Layer Centrifugation; SLC), making it quicker and easier to use than a density gradient, while affording the same degree of sperm selection (15). The major advantage of SLC is that it can be scaled up easily to process large volumes of semen, which would be challenging with a density gradient (16).

The SLC has been used previously to process buck, red deer and ram semen (17–19) but fertility was not examined in these studies. Therefore, the aim of the present study was a proof-of-concept, namely, to investigate whether SLC could be used to select robust ram spermatozoa that could survive cryopreservation whilst maintaining their fertilizing potential.

## 2 Methods

### 2.1 Animals

Mature rams of various breeds were identified as being suitable for sires in an artificial insemination program. With owner consent, they were transported to an EU-approved semen collection facility approximately 1 month prior to the start of the breeding season and were placed in isolation from other ruminants for 1 month. Serum samples were sent for testing for various diseases in accordance with the regulations for trade in semen within the European Union (20). After the collection period, rams returned to their farms of origin for natural mating during the breeding season.

### 2.2 Semen collection

Semen was collected by artificial vagina after introducing them to a teaser ewe. Two ejaculates were collected per session, at an interval of approximately 30 mins. The semen was immediately extended 1:1 in warm (30 °C) Andromed (Minitüb, Tiefenbach, Germany), while an initial assessment was carried out of sperm concentration and subjective motility. Sperm concentration was measured with a photometer (model SDM1; Minitüb International, Tiefenbach, Germany). Subjective motility was estimated using a phase-contrast microscope fitted with a warm stage set at 38 °C. Ejaculates with ≥70% motility in fresh semen were deemed suitable for freezing.

### 2.3 Substandard ejaculates

One of these males, a 2-year old Gotland Pelt ram, produced ejaculates of insufficient quality for freezing during the first 3 weeks of the collection period. Therefore, it was decided to try preparing any sub-standard ejaculates produced during the last week of the collection period by SLC, to determine whether sperm quality could be improved enough for cryopreservation. Five ejaculates identified as being < 70% motile in fresh semen were prepared by centrifugation through Ovicoll. Briefly, after adjusting the sperm concentration to 100 × 10^6^ spermatozoa/mL with Andromed, 20 ml of extended semen were layered over 15 ml Ovicoll in a 50 mL tube and centrifuged for 20 min at 300 g. The supernatant was removed and the sperm pellet was resuspended in Andromed before assessing sperm motility again. Initially, the sperm pellet was resuspended in 2 ml Andromed and the sperm concentration was measured using a Nucleocounter SP100 (Chemometic, Allerød, Denmark). If the sperm motility was ≥70% in the SLC sample, the sperm sample was frozen as described in the following section.

### 2.4 Semen freezing

The sperm concentration was subsequently adjusted to 500 × 10^6^ spermatozoa/mL with Andromed at room temperature (approximately 18 °C), packaged into 0.5 ml plastic straws and placed in the refrigerator at 6 °C for 2 h. The semen was then frozen in liquid nitrogen vapor in a programmable freezing machine (Icecube; Minitüb, Tiefenbach, Germany) before transferring to liquid nitrogen for storage. A post-thaw check was performed at least 24 h later; thawing occurred by placing the straws in a circulating water bath at 35 °C for 30 s and dispensing the contents into a warm tube in a heating block. Only samples with post-thaw motility of ≥50% were retained and later distributed for AI. Some lambing results from artificial insemination of the thawed semen was made available by the farmer. The artificial insemination was carried out on-farm by trained personnel, depositing thawed semen in the vagina of ewes in natural oestrus.

## 3 Results

Semen quality in ten ejaculates collected immediately prior to this trial ranged in motility from 25%−50% (mean ± SD 35.5 ± 7.6%). Five further ejaculates with motility ranging from 25%−50% were obtained in the last week of the semen collection period and were used for SLC. Sperm motility was improved after SLC (Table 1), enabling straws of semen to be frozen. All batches had acceptable post-thaw motility.

**Table 1:** Subjective sperm motility in sub-standard ram ejaculates at various stages during processing: initial, after Single Layer Centrifugation through Ovicoll, and post-thawing (*n* = 5).

Ejaculate					
	1	2	3	4	5
Initial motility (%)	50	50	25	20	30
Motility after SLC (%)	80	90	90	80	80
Motility after thawing (%)	50	50	50	50	50
Recovery rate (%)	41	42	46	58	61
No. straws	7	7	5	7	7

Straws were distributed to various farms for AI. In total, 22 ewes were inseminated on different farms, resulting in the birth of 46 lambs, of which 4 were still-born.

## 4 Discussion

Preparation of ram semen by SLC with Ovicoll resulted in selection of motile spermatozoa that could withstand cryopreservation. Although only a small number of straws were produced, the thawed spermatozoa were fertile when used for AI. To our knowledge, these are the first AIs to be conducted with Ovicoll-prepared sperm samples resulting in the birth of lambs, providing a proof-of-concept to link laboratory findings with application in the field. Previous studies used colloids to prepare thawed ram spermatozoa (19, 21) but not to prepare the fresh semen prior to cryopreservation, and the sperm samples were not used for AI.

In the study by Bergstein-Galan et al. (21), different types of colloid were used (Polyvinylpyrrolidone [PVP]-coated silica or silane-coated silica) in two volumes (1 ml or 4 ml) to prepare thawed ram semen. The silane-coated silica produced more of an improvement in sperm quality than the PVP-coated silica; the 1 ml volume of colloid was preferred to reduce costs. However, Atalla et al. (22) found that PVP-coated silica was better than swim-up for preparing fresh ram spermatozoa *in vitro* embryo production, with better sperm motility and normal morphology than the controls. More embryos were produced from the PVP-coated silica separation than from the swim-up treatment group.

Šterbenc et al. (19) processed ram semen by SLC after thawing, achieving better sperm quality in the SLC samples than in controls. Sperm motility, membrane integrity, normal morphology and acrosome integrity were all improved in the SLC-selected samples compared to controls, leading the authors to suggest that this method might aid initiatives for cryopreserving ram semen (19). Sperm fertility was not evaluated in their study.

Buck semen was processed by SLC either before or after freezing (17). Samples processed by SLC post-thawing showed improved sperm characteristics (sperm motility, membrane integrity, high mitochondrial membrane potential) compared to controls or samples where the SLC was done prior to freezing. There was no improvement in sperm quality from SLC before freezing compared to controls, in contrast to the present study where subjective motility was increased. However, the initial motility was lower in the present study with ram semen than in the buck ejaculates from the Spanish study and a different protocol was used for cryopreservation (17).

In a similar study with ejaculated semen from red deer (18), sperm motility, viability, mitochondrial membrane potential and acrosomal status were higher in SLC samples than controls. The SLC was performed post-thaw. Epididymal sperm samples recovered post-mortem were also tested. The authors concluded that the selection technique could be used with both ejaculated semen and epididymal sperm samples to assist reproductive technologies in this species.

The present results are in keeping with other studies involving species-specific colloid formulations for stallion and bull semen, in which robust spermatozoa could be selected from poor quality ejaculates. In stallions, the SLC-selected sperm samples derived from poor quality ejaculates were cooled and used for AI, resulting in pregnancies and the birth of offspring (23). In cattle, SLC was used to select robust spermatozoa from poor quality ejaculates from young bulls, resulting in post-thaw samples reaching the quality control thresholds of the breeding company (24). Determining fertility of the thawed SLC samples was not possible in the present study but, as shown, some information was available from the farms where the thawed semen was inseminated. These limited results show that the thawed sperm samples were able to fertilize oocytes after artificial insemination, which is encouraging and should pave the way for larger controlled studies.

The proportion of still-born lambs in this study was approximately 10% (4 out of 46), in keeping with other reports of 8%−30% still-births among lambs worldwide (25), although these global statistics may include deaths prior to weaning. However, in Norwegian flocks Holmøy and Waage (26) considered that 4% of lambs were still-born, considerably lower than the present study. Many factors contribute to still-birth, including genetics, number of lambs per pregnancy, production system, housing, flock size, and weather, among others (27).

Although this study is limited, the results are encouraging for further studies on cryopreserving ram semen. A larger trial is warranted, using split ejaculates to provide control and selected sperm samples for freezing, and incorporating several breeds of sheep. A previous report indicated that high pregnancy rates with thawed ram semen in Norwegian sheep breeds could be attributed to the anatomy of the cervix, which was different in these breeds (28). Still, the present results represent one step forward in this complex phenomenon of semen cryopreservation in sheep.

## 5 Conclusion

Sub-standard ram semen samples were processed by SLC with Ovicoll, resulting in improved sperm motility and subsequent cryosurvival. Using this technique, five ejaculates were frozen successfully, with acceptable post-thaw sperm motility. Although an AI trial was not intended to be part of this study, some *ad hoc* results showed that healthy lambs were produced after insemination of thawed sperm samples.

## Data Availability

The original contributions presented in the study are included in the article/supplementary material, further inquiries can be directed to the corresponding author.
